# Structure-Based Identification of SARS-CoV-2 nsp10-16 Methyltransferase Inhibitors Using Molecular Dynamics Insights

**DOI:** 10.3390/cimb47030198

**Published:** 2025-03-17

**Authors:** Ahmad M. Alharbi

**Affiliations:** Department of Clinical Laboratories Sciences, College of Applied Medical Sciences, Taif University, P.O. Box 11099, Taif 21944, Saudi Arabia; a.alharbii@tu.edu.sa

**Keywords:** SARS-CoV-2 nsp10-16, methyltransferase inhibition, molecular dynamics simulation, structure-based drug discovery

## Abstract

SARS-CoV-2 evades immune detection via nsp10-16 methyltransferase-mediated 2′-O-methylation of viral mRNA, making it a key antiviral target. Our study employed structure-based drug discovery—including virtual screening, molecular docking, and molecular dynamics (MD) simulations—to identify potent inhibitors of nsp10-16. We identified seven promising inhibitors (Z1–Z7) targeting the binding site of the SARS-CoV-2 nsp10-16 methyltransferase, with Z2, Z3, Z4, and Z7 exhibiting strong binding affinities. Further, molecular dynamics simulations confirmed that Z2, Z3, and Z7 effectively stabilized the enzyme by reducing conformational fluctuations and maintaining structural compactness, comparable to the native ligand-bound complex. The conformational deviation revealed that Z2, Z6, and Z7 restricted large-scale conformational transitions, reinforcing their stabilizing effect on the enzyme. The binding free energy calculations ranked Z4 (−37.26 kcal/mol), Z7 (−35.37 kcal/mol), and Z6 (−35.22 kcal/mol) as the strongest binders, surpassing the native tubercidin complex (−23.70 kcal/mol). The interactions analysis identified Asp99, Tyr132, and Cys115 as key stabilizing residues, with Z2, Z6, and Z7 forming high-lifetime hydrogen bonds. The drug-likeness analysis highlighted the selected compounds as promising candidates, exhibiting high gastrointestinal absorption, optimal solubility, and minimal CYP450 inhibition. Further experimental validation and lead optimization are needed to develop potent methyltransferase inhibitors with improved pharmacokinetics and antiviral efficacy.

## 1. Introduction

Severe acute respiratory syndrome coronavirus 2 (SARS-CoV-2), a positive-sense RNA virus belonging to the family *Coronaviridae*, caused a global pandemic with substantial mortality and morbidity [1]. Its replication machinery includes several non-structural proteins (nsps) critical for viral RNA synthesis and post-transcriptional modifications. Among these, the heterodimeric complex of nsp10 and nsp16 performs a vital function in viral mRNA capping by catalyzing the 2′-O-methylation of the ribose moiety at the 5′ cap [2]. This modification converts the Cap0 structure into Cap1, mimicking host mRNA and preventing recognition by innate immune sensors such as MDA5 and IFIT [3]. The enzymatic activity resides in nsp16, while nsp10 acts as an activator that stabilizes the catalytic site [4]. Failure to methylate the viral mRNA cap results in translational inhibition and immune activation, emphasizing the critical role of this complex in viral survival and pathogenesis [5]. Given its essential role in viral replication and immune evasion, the nsp10-16 complex has emerged as a promising antiviral target. Unlike other RNA methyltransferases, nsp16 exhibits a unique structural architecture with a large S-adenosylmethionine (SAM) binding pocket that offers opportunities for selective inhibitor design [6]. Despite structural knowledge of the nsp10-16 complex and its interactions with various cofactors, effective small-molecule inhibitors targeting this enzyme remain scarce. Consequently, the identification and development of specific inhibitors against nsp10-16 could provide novel therapeutic options for COVID-19 and related coronavirus infections [7].

Despite the therapeutic potential of nsp10-16 as an antiviral target, the development of selective inhibitors has been challenging due to the structural and functional complexity of this enzyme complex [8,9]. The active site of nsp16 accommodates SAM as a methyl donor for 2′-O-methylation, making the SAM-binding pocket a primary target for small-molecule inhibitors [10]. However, achieving selectivity for viral methyltransferases over human methyltransferases—which also utilize SAM as a cofactor—is difficult due to the conserved nature of SAM-binding motifs [11]. Although several SAM analogs, such as sinefungin, have shown inhibitory activity against nsp10-16 in vitro, they suffer from poor cellular permeability and low specificity, limiting their therapeutic efficacy [12]. Additionally, inhibitors like tubercidin, a known nucleoside analog, have demonstrated broad-spectrum antiviral activity but exhibit significant cytotoxicity due to off-target effects on host methyltransferases [7]. Moreover, the enzyme’s conformational flexibility further complicates inhibitor design. Structural studies have revealed that the nsp10-16 complex undergoes dynamic changes upon ligand binding, particularly in the gate loop regions that regulate access to the SAM-binding site [13]. These conformational changes can influence inhibitor binding and efficacy [14]. Therefore, an effective inhibitor must not only bind tightly to the target site but also induce conformational changes that disrupt the enzyme’s catalytic function. Given these challenges, there is a pressing need for rational drug design approaches that exploit the unique structural features of nsp10-16 while minimizing off-target interactions with host methyltransferases [15].

Given the structural complexities and challenges associated with targeting nsp10-16, a structure-based drug-discovery approach offers a promising pathway for identifying selective inhibitors [16]. Structure-based methods use detailed knowledge of the enzyme’s three-dimensional conformation, allowing the design and optimization of molecules that can fit precisely into the SAM-binding pocket or other potential binding sites [17]. Recent advances in X-ray crystallography and computational docking have provided high-resolution structures of nsp10-16 in complex with various ligands, including SAM, sinefungin, and tubercidin derivatives [7]. These structures have revealed critical binding interactions and conformational changes, offering valuable insights into the design of novel inhibitors [18]. In silico screening of large compound libraries against the SAM-binding site can rapidly identify potential lead molecules that exhibit favorable binding profiles [19]. Molecular docking and dynamic simulations further enable the evaluation of binding stability, flexibility, and protein–ligand interactions under near-physiological conditions [20]. Additionally, pharmacokinetic modeling helps assess drug-like properties, such as absorption, distribution, metabolism, and toxicity, early in the design process [21]. By integrating computational screening, molecular dynamics, and in vitro assays, structure-based drug discovery not only accelerates the identification of promising candidates but also reduces the likelihood of late-stage failure due to poor efficacy or safety [22].

The present study aims to identify novel inhibitors of the SARS-CoV-2 nsp10-16 methyltransferase complex through a structure-based drug-discovery approach. While this study focuses on identifying binding inhibitors for SARS-CoV-2 nsp10-16 methyltransferase, achieving selectivity is critical as human methyltransferases also utilize SAM as a cofactor and share conserved SAM-binding motifs. This structural similarity increases the challenge of developing inhibitors that are specific to the viral enzyme without affecting human proteins. Therefore, this work focuses on viral specificity to ensure both efficacy and safety. Utilizing the high-resolution crystal structure of the nsp10-16 complex bound to tubercidin, we conducted virtual screening of a large compound library to identify potential inhibitors targeting the SAM-binding site. To ensure viral specificity, we prioritized compounds interacting with residues unique to SARS-CoV-2 nsp16, such as Asp99, Tyr132, and Cys115, while minimizing interactions with human methyltransferases. Since docking was performed using a rigid protein structure, the binding site was pre-formed; however, molecular dynamics simulations were subsequently employed to assess protein flexibility and validate ligand stability in a dynamic environment. The top-ranked compounds were further evaluated for their drug-likeness and pharmacokinetic profiles. To ensure the stability and efficacy of the selected compounds, molecular dynamics simulations were performed, followed by binding free energy calculations to quantify their interaction strength with the enzyme. Our work not only identifies promising lead compounds but also provides a framework for future development of specific inhibitors targeting viral RNA methyltransferases.

## 2. Materials and Methods

### 2.1. Protein Crystal Structure Retrieval

The X-ray diffraction structure of the SARS-CoV-2 nsp10-16 methyltransferase complex with tubercidin was retrieved from the RCSB Protein Data Bank (https://www.rcsb.org/, accessed on 30 January 2025) with PDB ID: 8BSD [7]. Molecular Operating Environment (MOE) version 2024.06 [23] was employed to prepare and refine the protein structure. The Loop Modeler algorithm, in conjunction with the Amber14–EHT forcefield (Amber ff14SB combined with the Extended Hückel Theory) [24,25], was used to model any missing residues in the three-dimensional (3D) structure of the protein. The QuickPrep tool in MOE was utilized to add missing hydrogen atoms to the protein residues and to assign appropriate charges to the terminal residues (C-terminal and N-terminal) of the nsp10-16 methyltransferase complex. Missing parameters in the forcefield—including atom types, van der Waals interactions, bond angles, and the chirality of residues—were checked and corrected to ensure a complete and accurate 3D protein structure.

### 2.2. Molecular Docking Simulation

The binding modes of ZINC20 in-stock (https://zinc20.docking.org/, accessed on 30 January 2025) compounds within the tubercidin-binding site of the SARS-CoV-2 nsp10-16 methyltransferase complex were predicted using the dock application of MOE 2024.06. To ensure the reliability of the docking protocol, a validation step was performed by re-docking the co-crystallized inhibitor tubercidin into its original binding site. The accuracy of the protocol was evaluated by calculating the root mean square deviation (RMSD) between the co-crystallized and re-docked inhibitor, ensuring that the RMSD was below 2.0 Å. The docking protocol comprised three major phases. First, the ligand geometry was evaluated using the ligand preparation tool in MOE, where the structure of each ligand was optimized through energy minimization. The process involved optimizing bond lengths, angles, and torsional degrees of freedom to achieve a reasonable conformational space for docking. The energy minimization was carried out using the Amber14–EHT force field, ensuring that the ligands adopt energetically favorable conformations before docking. The protonation state of all ligands was assigned based on the intracellular pH (~7), considering the pKa values of the ligands’ titratable groups. Next, maximum ligand conformations were generated using the Triangle Matcher algorithm as the placement method, retaining 30 conformations per ligand. The binding energies for each conformation were estimated using the London dG scoring function, which computes binding free energy based on the following equation:

(1) $$G=c+E_{flex}+\sum_{h-bonds}c_{HB}f_{HB}+\sum_{m-lig}c_{M}f_{M}+\sum_{atom\;i}∆D_{i}$$

where c represents the changes in entropy due to rotational or translational freedom; E_flex_ is the ligand’s flexibility energy loss; f_HB_ accounts for H-bond imperfections; C_HB_ denotes the energy of favorable hydrogen bonds; f_M_ represents metal–ligand imperfections; while C_M_ and D_i_ compute metal-binding and desolvation energies, respectively. The final refinement of the docked ligand conformations was carried out using the GBVI/WSA dG scoring function (Equation (2)) in MOE. In this phase, the top 10 docked conformations were subjected to energy minimization while maintaining the protein structure as fixed. Protein flexibility was not explicitly incorporated in this phase. However, flexibility was considered indirectly by evaluating the docking poses based on their binding affinity and stability in the active site. RMSD values were calculated to assess the consistency of ligand poses before and after the refinement phase.

(2) $$∆G\approx c+a\left[\frac{2}{3}\left(∆E_{coul}+{∆E}_{sol}\right)+∆E_{vdw}+\beta ∆SA_{weighted}\right]$$

where c denotes the changes in entropy; α and β are constants determined by the selected forcefield; E_coul_ and E_sol_ represent coulombic and solvation energies, respectively; E_vdW_ represents van der Waals contributions; and SA_weighted_ denotes the weighted solvent-accessible surface area.

The ZINC20 in-stock database, comprising 12,357,725 drug-like compounds in SDF format, was obtained from the ZINC20 server and converted to MOE’s MDB format using the MOE database viewer. The initial filtering of the ZINC20 in-stock database was performed based on molecular size and the removal of duplicate structures. These compounds were docked into the methyltransferase’s active site using the validated docking protocol. The resulting docked complexes (saved in PDB format) were analyzed for protein–ligand interactions using the Protein–Ligand Interaction Fingerprinting (PLIF) tool in MOE, which provides a detailed profile of hydrogen bonds, π-π interactions, and metal–ligand coordination.

### 2.3. Estimation of Pharmacokinetic, Drug-Likeness, and Physicochemical Profiles

To evaluate the pharmacokinetic properties and safety profile of the final selected ZINC20 in-stock compounds, the absorption, distribution, metabolism, excretion, and toxicity (ADMET) parameters were predicted using the Deep-PK server [26], which employs graph neural networks for accurate modeling. The evaluation encompassed key pharmacokinetic characteristics—including absorption, distribution, metabolism, excretion, and toxicity. Drug absorption was predicted using permeability models based on human colorectal adenocarcinoma (Caco-2) cells and Madin–Darby Canine Kidney (MDCK) cells. Distribution assessments were conducted by estimating the compounds’ ability to penetrate the blood–brain barrier (BBB), a critical factor for central nervous system drugs. Metabolism predictions focused on interactions with major cytochrome P450 (CYP450) enzymes, enabling the identification of potential metabolic pathways and risks of metabolic instability. Excretion metrics, including drug clearance and half-life, were calculated to predict elimination rates.

### 2.4. Molecular Dynamics Simulations

Molecular dynamics (MD) simulations provide critical insights into the dynamic behavior of biomolecular systems, particularly in understanding protein–ligand interactions, which is essential for rational drug design [27]. In our study, the MD simulations were performed on the SARS-CoV-2 nsp10-16 methyltransferase complex (PDB ID: 8BSD) in both its unbound (apo) form and when bound to seven inhibitor compounds (Z1–Z7) selected from the ZINC20 database after screening and docking. Additionally, the apo-form and the native co-crystallized tubercidin-bound complex (8BSD) were used as control systems. The simulations were conducted using AMBER24 software(version 24) [28], with an explicit solvent model applied to simulate the aqueous environment surrounding the protein–ligand complexes. System setup was performed using the AMBER24 LEaP module, applying the ff19SB force field for protein residues [29]. Topology and coordinate files were generated for all protein systems, and each system was solvated in a truncated octahedral box filled with OPC water molecules, ensuring a 12-angstrom buffer around the protein. Electrostatic neutrality was maintained by adding Na^+^ and Cl^−^ ions at a 0.1 M concentration [30]. The force field parameters for the ligands were derived using the Generalized Amber Force Field version 2 (GAFF2) for ligand parameterization. To ensure accuracy, HF/6-31G level quantum mechanical (QM) calculations were employed for structure preparation and charge assignment during AM1-BCC charge calculations [31]. Missing hydrogen atoms were added to the structures, and the Parmchk2 tool was used to assign appropriate force field parameters for small molecules [32]. To ensure proper hydrogen positioning and maintain structural stability, the SHAKE algorithm was applied to constrain all bonds involving hydrogen atoms, allowing a time step of 2 femtoseconds (fs) [33]. Long-range electrostatic interactions were calculated using the particle-mesh Ewald (PME) method, and the simulations were optimized for GPU-based execution to enhance computational efficiency [34]. The initialization of MD simulations involved a two-step energy minimization process. The first step consisted of 50,000 steps of steepest descent minimization, followed by 25,000 steps of conjugate gradient minimization, with harmonic restraints applied to the protein [35]. After minimization, the systems were gradually heated from 0.1 K to 300 K over 500 picoseconds (ps) using an NVT ensemble and a Langevin thermostat for temperature control [36]. Density equilibration was performed by gradually adjusting the system’s density under constant volume. Once the target temperature was reached, equilibration was continued using an NPT ensemble for 10,000 ps without restraints, maintaining constant pressure via isotropic position scaling with a relaxation time of 2 ps [36]. Production MD simulations were carried out for 200 nanoseconds (ns) in the NPT ensemble at 300 K, with a trajectory snapshot recorded every 20 ps, we used the Langevin thermostat for temperature control at 300 K, and the Monte Carlo barostat was applied to maintain constant pressure at 1 atm. The electrostatic cutoff was set to 8 Å for non-bonded interactions.

### 2.5. Protein Stability Assessment

The stability of the SARS-CoV-2 nsp10-16 methyltransferase was evaluated by analyzing the root mean square deviation (RMSD) of the Cα atoms throughout the 200-nanosecond (ns) MD simulation using the CPPTRAJ module of AMBER24 [37]. The RMSD calculation used the initial frame of each trajectory as the reference and a total of 10,000 frames were analyzed to track deviations over time. This provided a detailed profile of structural changes and fluctuations, enabling the identification of stable or unstable regions during the simulation. The average RMSD values were computed to compare the structural stability of the unbound protein (apo), the control system (8BSD complex), and the seven selected inhibitor-bound complexes.

### 2.6. Amino Acid Fluctuations and Structural Density Analysis

To examine the flexibility of individual residues in the nsp10-16 methyltransferase, root mean square fluctuation (RMSF) analysis was performed using the CPPTRAJ module [37]. RMSF values were calculated for each residue to quantify its movement relative to its average position over the simulation time. This RMSF helped in identifying flexible and rigid regions within the protein, which are crucial for understanding ligand-binding effects.

In addition, the radius of gyration (RG) was computed to assess the overall structural compactness of the protein during the simulation. The RG measures the distribution of the protein’s mass around its center of mass, and changes in RG values indicate alterations in protein folding and packing density. The RG was calculated using CCPTRAJ and monitored for both the unbound (apo) protein and the inhibitor-bound complexes across the 200-nanosecond trajectory to explore how ligand binding affects the protein’s structural integrity and compactness [37].

### 2.7. Principal Component Analysis (PCA)

To understand large-scale conformational changes in the protein during the MD simulations, principal component analysis (PCA) was performed. PCA identifies major collective motions within the protein by analyzing its trajectory in three-dimensional space [38]. The covariance matrix of atomic displacements was generated from the MD trajectory, and orthogonal eigenvectors (principal components) were computed to represent the predominant motions. The first three principal components, which account for the highest variance in structural motion, were analyzed in detail. By projecting the trajectory onto 10 components, we visualized the dominant conformational states and their transitions over time. Additionally, K-means clustering was applied to the projected PCA data, grouping similar conformations based on their RMSD values that helped to identify major conformational states and shifts in the nsp10-16 methyltransferase upon ligand binding, exploring its dynamic behavior.

### 2.8. Hydrogen Bond Analysis

Hydrogen bonds (H-bonds) play a critical role in maintaining protein stability and mediating protein–ligand interactions. Therefore, H-bond analysis was conducted using the CPPTRAJ module of AMBER24 to quantify the number and stability of H-bonds formed during the simulations [39]. The analysis focused on identifying key residues involved in H-bond formation in both the apo form and inhibitor-bound complexes. H-bonds were identified based on a donor–acceptor distance threshold of ≤3.5 Å and a minimum bond angle of 120°. The stability of H-bonds was determined by calculating their occupancy percentage over the 200-nanosecond simulation. H-bonds in the active site of the protein were examined in detail, particularly those involving residues that directly interact with the inhibitors as they are crucial for understanding binding stability and specificity [37].

### 2.9. Binding Free Energy Calculations

Binding free energy (BFE) calculations were performed to quantify the strength of interaction between the nsp10-16 methyltransferase and the selected inhibitors. For this, the Molecular Mechanics/Generalized Born Surface Area (MM/GBSA) method was employed using the last 2500 frames (~50 ns) of each production trajectory [40,41,42,43,44]. The binding free energy was calculated using the following equation [45]:

(3) $$\Delta G_{bind}={\Delta G}_{R+L}-\left(\Delta G_{R}+{\Delta G}_{L}\right)$$

where ΔG_bind_ is the binding free energy; ΔG_R+L_ is the free energy of the protein–ligand complex; and ΔG_R_ and ΔG_L_ are the free energies of the receptor and ligand, respectively.

The MM/GBSA calculations decomposed the total free energy into its individual components, including bond energy (E_bond_), van der Waals energy (E_vdW_), electrostatic energy (E_elec_), and solvation energy. Solvation energy was further divided into polar (G_GB_) and non-polar (G_SA_) components, where the polar solvation energy was estimated using the Generalized Born (GB) model, and the non-polar solvation energy was approximated using the solvent-accessible surface area (SASA) method. The equation used for calculating the total free energy is given below:

(4) $$G=E_{bond}+E_{VDW}+E_{elec}+G_{GB}+G_{SA}-TS_{S}$$

where TSS represents the vibrational entropy from the second derivative of the potential energy surface, capturing contributions from protein and ligand flexibility. The BFE values were reported in kcal/mol for all eight systems, the control complex (8BSD), and the seven inhibitor-bound complexes to provide quantitative insights into the binding affinities of the selected inhibitors, aiding in the identification of the most promising candidates for further development.

### 2.10. Data Examination and Visualization

Detailed structural illustrations were prepared using MOE2024.06 (version 2024) [23] and CueMol, enabling accurate visualization of the protein–ligand complexes. Graphical representations of key parameters—including the RMSD, RMSF, radius of gyration (RG), and PCA results—were generated using OriginPro (version 9.0) to facilitate clear and precise interpretation of the dynamic behavior and interactions observed during the MD simulations [46].

## 3. Results and Discussion

### 3.1. Protein Crystal Structure

**Figure 1:**
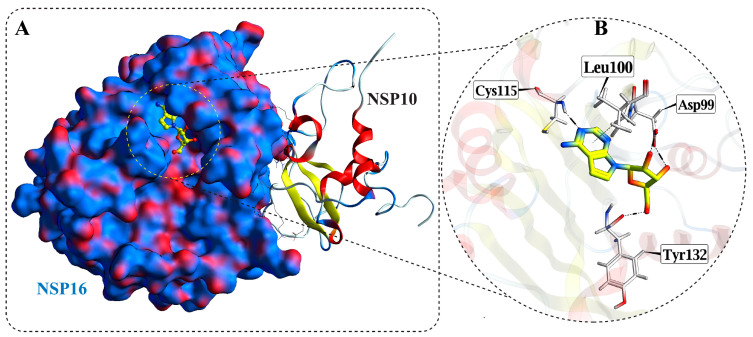
Crystal structure of the SARS-CoV-2 nsp10-16 methyltransferase complex and key interactions in the active site: (**A**) Surface representation of the nsp16 and nsp10 (cartoon) complex, with tubercidin (yellow) bound in the active site. The catalytic pocket where the ligand binds is highlighted. (**B**) Close-up view of the tubercidin binding site showing critical residues involved in stabilizing the ligand, which form key hydrogen bonds and hydrophobic interactions with tubercidin.

The high-resolution crystal structure of the SARS-CoV-2 nsp10-16 methyltransferase complex in association with tubercidin (PDB ID: 8BSD) was selected for our study (Figure 1). This structure provides a well-defined representation of the enzyme’s catalytic region and the SAM-binding pocket. Tubercidin, a nucleoside analog known for its antiviral properties, was co-crystallized in the active site, mimicking natural substrate binding. The nsp10-16 methyltransferase complex consists of the catalytic subunit nsp16 and its cofactor nsp10, which stabilizes the enzyme’s conformation and enhances its 2′-O-methylation activity. Detailed analysis of the crystal structure revealed key interactions between tubercidin and residues within the active site of nsp16. Tubercidin forms hydrogen bonds with several critical residues, stabilizing its binding. Specifically, the O5 atom of tubercidin interacts with the main chain carbonyl of TYR132 through a hydrogen bond at a distance of 2.5 Å, contributing an energy of −2.0 kcal/mol. Additionally, the ligand O2 and O3 atoms form hydrogen bonds with ASP99, with bond distances of 2.5 Å and 2.7 Å, respectively, contributing energies of −4.0 kcal/mol and −3.6 kcal/mol, respectively. Another important interaction of the tubercidin is the formation of a hydrogen bond with the side chain sulfur of CYS115 at a distance of 3.0 Å, contributing −4.0 kcal/mol to the binding energy. These interactions are critical for stabilizing tubercidin within the active site, suggesting its potential as a competitive inhibitor by mimicking the native SAM substrate. The strong hydrogen bonding network observed between tubercidin and residues TYR132, ASP99, and CYS115 underscores the structural basis for its inhibitory activity. These results form the basis for subsequent computational screening aimed at identifying other molecules capable of forming similar and stronger interactions with the nsp10-16 methyltransferase complex.

### 3.2. Molecular Docking Simulations

**Figure 2:**
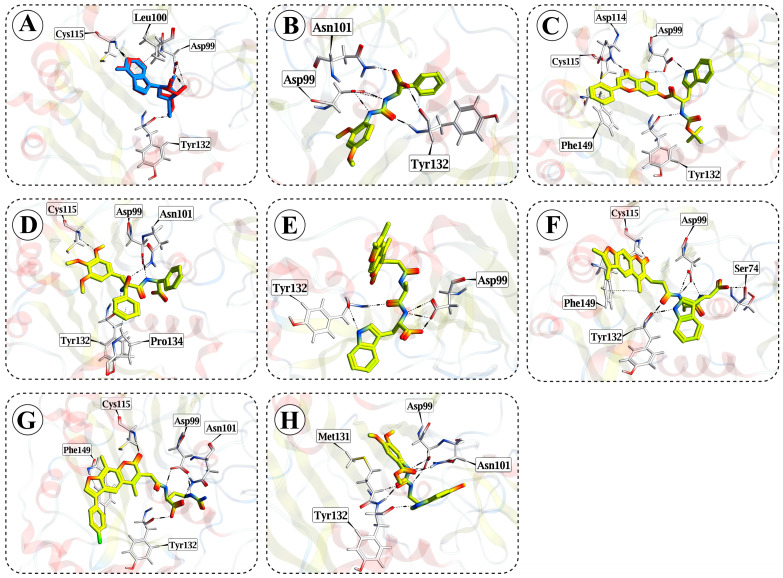
Docking validation and compound poses: (**A**) superimposition of the re-docked tubercidin (red) with the co-crystalized ligand (blue) from the PDB structure (ID: 8BSD), with an RMSD value of 0.517 Å, demonstrating the accuracy of the docking protocol; (**B**–**H**) docking poses of the compounds Z1 to Z7 in the protein binding site.

The virtual screening of drug-like compounds targeting the SAM-binding site of the SARS-CoV-2 nsp10-16 methyltransferase was carried out using a structure-based docking approach. A total of 12,357,725 drug-like compounds from the ZINC20 database were initially screened to identify potential inhibitors based on binding affinity and interaction profiles. The docking protocol was validated through re-docking of the co-crystallized inhibitor tubercidin, which reproduced the native binding pose with an RMSD of 0.517 Å, confirming the accuracy and reliability of the docking method. From the initial screening, compounds were ranked by their binding energies, and the top-ranking hits were further analyzed based on their ability to form key interactions with critical residues in the active site. After multiple rounds of refinement, seven compounds with the most favorable binding profiles were selected for further studies (ZINC IDs: ZINC544166, ZINC2087193, ZINC2109321, ZINC2111032, ZINC2111034, ZINC2112495, and ZINC2112958) (Figure 2). The docking scores and refined RMSD values for the seven selected compounds (Z1–Z7) targeting the SAM-binding pocket of the SARS-CoV-2 nsp10-16 methyltransferase are presented in Table 1. Z1 (ZINC544166) exhibited a high docking score, suggesting strong binding affinity. However, its refined binding pose showed moderate stability, with some positional deviation. Despite this, Z1 formed key hydrogen bonds with critical residues, such as Tyr132 and Asp99, which likely contributed to its favorable docking score. Z2 (ZINC2087193) and Z3 (ZINC2109321) also displayed high docking scores, indicating strong binding affinity. Notably, both compounds maintained highly stable binding poses after refinement, closely resembling their initial docked conformations. The presence of multiple methoxy groups in both Z2 and Z3 likely facilitated hydrophobic interactions with key residues in the active site, further supporting their high docking scores. Z4 (ZINC2111032) demonstrated not only a high docking score but also exhibited the most stable refined binding pose among the compounds tested. This stability suggests effective interactions with key residues in the SAM-binding site. The aromatic ring system and ester group in Z4 likely contributed to favorable hydrophobic and hydrogen bond interactions, reinforcing its stable and favorable binding. Z5 (ZINC2111034) showed a high docking score as well, though its binding pose was moderately stable after refinement. The fluorine-substituted aromatic system in Z5 may enhance binding through π-π stacking interactions with aromatic residues within the binding pocket, contributing to its overall binding affinity. Z6 (ZINC2112495) and Z7 (ZINC2112958) both exhibited high docking scores but demonstrated less stability in their refined binding poses compared to the other compounds. These results suggest that, while the compounds interact favorably with the active site, their binding poses were less consistent following refinement. Despite their lower binding affinities compared to the other compounds, they maintained interactions with key residues in the active site. Z6 features a flexible aliphatic chain, which may have contributed to its higher RMSD-refined value. Z7 contains methoxy groups on its aromatic ring, facilitating weak hydrogen bonding and hydrophobic interactions. Overall, compounds Z2, Z3, and Z4 exhibited the most favorable combination of docking scores and low RMSD-refined values, suggesting that they are the most promising candidates for further investigation. Their stable binding poses and strong interactions with key active site residues, including Tyr132, Asp99, and Cys115, highlight their potential as inhibitors of the nsp10-16 methyltransferase complex.

### 3.3. Molecular Interactions

The binding interactions of the seven selected compounds (Z1–Z7) with key residues of the SAM-binding pocket of SARS-CoV-2 nsp10-16 methyltransferase were analyzed (Figure 1; Supplementary Materials, Table S1). Each compound exhibited significant hydrogen bonding and hydrophobic interactions with essential residues, including Tyr132, Asp99, Cys115, Asn101, and Phe149, crucial for ligand stabilization in the SAM-binding site. Z1 formed strong hydrogen bonds with Asp99, Tyr132, and Asn101, with binding energies ranging from −0.5 kcal/mol to −6.6 kcal/mol, while Z2 interacted with Tyr132, Asp99, Asp114, and Cys115 showing additional π-H interactions with Phe149. Z3 displayed significant interactions with Tyr132, Asp99, Cys115, and Asn101, with particularly strong binding to Asp99 (binding energy of −6.2 kcal/mol). Z4 showed hydrogen bonds with Asp99 and Tyr132, contributing to its binding stability, while Z5 exhibited key interactions with Asp99, Tyr132, and Cys115, with a particularly strong hydrogen bond to Tyr132 (binding energy of −6.1 kcal/mol). Z6 demonstrated the strongest binding, with interactions involving Asp99, Tyr132, Cys115, and Asn101, including hydrogen bonds with Asp99 at 2.8 Å and 3.0 Å (binding energies of −6.9 kcal/mol and −3.5 kcal/mol, respectively). Z7 formed interactions with Asp99, Tyr132, Asn101, and Met131, with energy contributions from hydrogen bonds and π-H interactions with Phe149. In comparison to tubercidin, all compounds showed similar interactions with Tyr132, Asp99, and Cys115 but many also engaged with additional residues such as Asn101, Asp114, and Pro134; however, while these residues may contribute to selectivity, additional comparative binding studies with homologous human methyltransferases are necessary to confirm their role in specificity. The reanalysis, focusing on nsp16-specific residues near the SAM-binding site, further highlighted Z3 and Z6 as the most promising inhibitors due to their stronger and more selective binding interactions, which may offer better specificity for nsp16 over other SAM-dependent enzymes.

### 3.4. ADMET and Drug-Likeness Analyses of the Selected Compounds

The selected compounds (Z1–Z7) were assessed through ADMET analysis to determine their drug-likeness, pharmacokinetic properties, and safety profiles. Key molecular descriptors such as molecular weight, number of heavy atoms, and rotatable bonds are summarized in Table S2. The compounds exhibited varying molecular weights, with most falling within the drug-likeness threshold. While Z1, Z4, and Z6 exceeded this threshold slightly, their high fraction of sp3-hybridized carbons indicated favorable solubility and permeability. In terms of rotatable bonds, Z2 and Z7 had the fewest, which is generally favorable for oral bioavailability. Drug-likeness was further evaluated using Lipinski, Ghose, Veber, Egan, and Muegge rules (see Table S3). Z2, Z3, and Z7 exhibited no violations of these rules, indicating good oral bioavailability, while Z1, Z4, Z5, and Z6 showed minor violations related to molecular weight and rotatable bonds. Importantly, none of the compounds triggered PAINS alerts, suggesting a low risk of assay interference. Solubility predictions, as shown in Table S4, revealed that Z7 had the highest solubility while Z4 and Z6 exhibited lower solubility. Gastrointestinal absorption potential indicated that Z2, Z3, and Z7 had high absorption, while the others displayed moderate absorption. Notably, none of the compounds were predicted to cross the blood–brain barrier, which reduces the likelihood of central nervous system-related side effects. Lipophilicity values, detailed in Table S5, indicated that Z1, Z4, and Z6 exhibited moderate-to-high lipophilicity, which could affect solubility and clearance. In contrast, Z7 displayed the lowest consensus LogP, suggesting a balanced lipophilicity favorable for both solubility and permeability. Cytochrome P450 (CYP) inhibition predictions, summarized in Table S6, revealed that Z2, Z3, and Z5 showed potential for inhibiting multiple CYP isoforms, while Z7 exhibited no inhibition, suggesting a lower potential for metabolic interactions. Z1 and Z6 showed selective CYP inhibition. Our results show that Z2, Z3, and Z7 displayed favorable ADMET profiles, with high gastrointestinal absorption, moderate lipophilicity, and good solubility. Among these, Z7 emerged as the most promising candidate, offering an optimal balance of properties, including excellent solubility, high GI absorption, and no CYP inhibition. Although Z1, Z4, Z5, and Z6 showed some limitations, their profiles suggest potential for further optimization.

### 3.5. MD Simulation Stability Analysis

**Figure 3:**
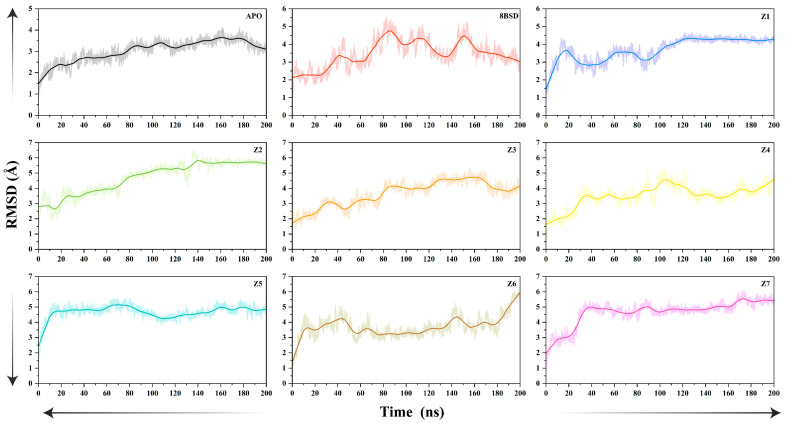
The root mean square deviation (RMSD) of SARS-CoV-2 nsp10-16 methyltransferase Cα atoms over 200-nanosecond simulation time for the unbound enzyme (apo), the native complex with tubercidin (8BSD), and seven selected inhibitor-bound systems (Z1–Z7).

To evaluate the structural stability of the SARS-CoV-2 nsp10-16 methyltransferase in its unbound (apo), native (8BSD), and inhibitor-bound forms, molecular dynamics (MD) simulations were conducted over a 200-nanosecond timescale. The root mean square deviation (RMSD) of the Cα atoms was calculated to monitor the overall conformational changes and stability of each system during the simulation, as shown in Figure 3. The apo system exhibited the highest RMSD fluctuations throughout the simulation, with values rising steadily and reaching approximately 4 Å by the end of the 200-nanosecond simulation. This indicates significant conformational flexibility and instability in the absence of a ligand, suggesting that the nsp10-16 methyltransferase alone undergoes considerable structural rearrangements in solution. In contrast, the 8BSD complex with tubercidin bound in the active site displayed relatively lower RMSD fluctuations, stabilizing around 3.5 Å after 160 ns and maintaining this value for the remainder of the simulation. The presence of tubercidin significantly stabilized the nsp10-16 methyltransferase complex, reducing structural fluctuations and maintaining enzyme integrity throughout the simulation. Among the seven inhibitor-bound systems, Z1 exhibited moderate stability, with RMSD values stabilizing at ~4.0 Å after 100 ns, indicating restricted conformational flexibility upon binding. Z2 and Z3 demonstrated enhanced stability, with RMSD values converging at ~6.0 Å and 4.0 Å, respectively, after 80 ns, suggesting effective enzyme–ligand interactions. Z4 exhibited a gradual RMSD increase, reaching ~3.5 Å by the end of the simulation, indicative of higher conformational flexibility despite initial stable binding. Conversely, Z5, Z6, and Z7 maintained consistent RMSD values ranging from 4.5 Å to 5.0 Å, demonstrating sustained structural integrity. Among these, Z7 exhibited the lowest overall RMSD (~5.0 Å), suggesting a strong stabilizing effect on the nsp10-16 complex, likely driven by persistent interactions within the active site. Comparison with the 8BSD–tubercidin complex revealed that most inhibitors (Z2, Z3, Z5, Z6, and Z7) exhibited comparable or slightly elevated RMSD values, indicating effective stabilization of the enzyme. The apo system exhibited RMSD drift, underscoring the necessity of ligand binding for maintaining the enzyme’s structural integrity. Notably, while Z2 displayed a higher RMSD (~6.0 Å), its fluctuation profile was more stable than the apo structure, suggesting that it reinforces enzyme stability despite minor conformational shifts. The relatively low RMSD values of Z3 and Z7 further indicate their potential to mimic tubercidin’s stabilizing effect on the nsp10-16 complex, making them strong candidates for further optimization and experimental validation. In summary, the MD simulation results indicate that inhibitors Z2, Z3, and Z7 provide the best stabilization of the nsp10-16 methyltransferase, comparable to or slightly exceeding the stability observed in the native 8BSD complex. These findings support the potential of Z2, Z3, and Z7 as promising candidates for further optimization and experimental validation.

#### Residue Fluctuation Analysis

**Figure 4:**
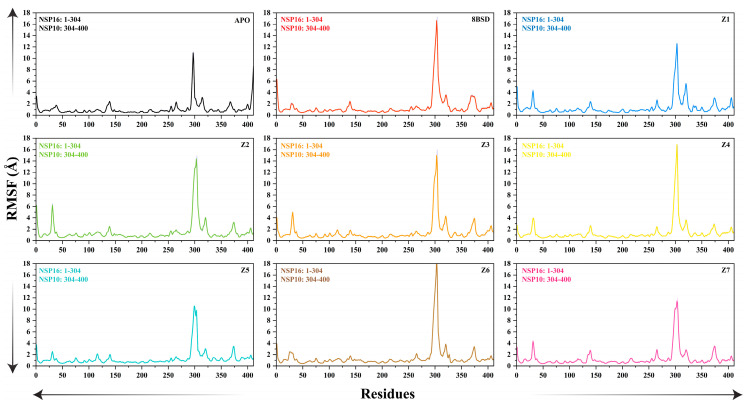
The root mean square fluctuation (RMSF) of SARS-CoV-2 nsp10-16 methyltransferase Cα atoms for the apo form, the native complex with tubercidin (8BSD), and the seven inhibitor-bound systems (Z1–Z7) over a 200-nanosecond simulation time.

Root mean square fluctuation (RMSF) analysis was performed using the CPPTRAJ module of AMBER24 to examine the flexibility of individual residues in the SARS-CoV-2 nsp10-16 methyltransferase complex. To improve clarity, the RMSF analysis was separated for nsp10 and nsp16, with each protein’s fluctuations shown independently in Figure 4. The starting and ending residues for each protein are clearly labeled in the plot. RMSF values greater than 10 Å are observed in regions of the proteins that exhibit higher flexibility, particularly in the N- and C-terminal regions, which are more flexible and likely to show diffusive motion. These high values reflect the natural dynamic behavior of these regions, rather than harmonic motion, and should be interpreted as indicative of their flexible nature in the absence of ligand binding. In the apo system, significant fluctuations were observed in several regions, with a prominent peak around residue 300, indicating high flexibility in this region when the protein is unbound. This region corresponds to the loop near the SAM-binding pocket, which may undergo conformational rearrangements in the absence of a ligand. The presence of such large fluctuations suggests that the apo form lacks structural stability, particularly in regions critical for ligand binding. In contrast, the 8BSD complex exhibited reduced fluctuations overall, with a higher RMSF peak around residue 300 (~16 Å), indicating that the binding of tubercidin stabilized the flexible C-terminal region. The increased flexibility suggests that ligand binding helps maintain a more flexible and stable conformation, particularly in areas involved in enzymatic activity and ligand interactions. Among the inhibitor-bound systems, Z2, Z3, and Z7 showed lower overall fluctuations, with RMSF peaks around residue 300, similar to the 8BSD complex (~9–12 Å). This suggests that these inhibitors effectively stabilize the C-terminal region, similar to tubercidin. Additionally, minor fluctuations were observed in the N-terminal and C-terminal regions, which are generally less critical for binding and enzymatic activity. Z1, Z4, Z5, and Z6 exhibited slightly higher RMSF peaks (~12–16 Å) around residue 300, indicating moderate flexibility in the C-terminal region. However, the fluctuations were significantly lower compared to the apo system, suggesting that ligand binding provides some degree of stabilization. Notably, Z4 and Z6 displayed additional minor peaks in other loop regions, suggesting that while they bind effectively, they may not restrict conformational flexibility as well as Z2, Z3, and Z7. Overall, the RMSF analysis highlights the stabilizing effect of ligand binding on the SARS-CoV-2 nsp10-16 methyltransferase, particularly in the active site region. The apo form showed a moderate degree of residue fluctuation, indicating inherent instability in the absence of a ligand. The 8BSD complex and the inhibitor-bound systems, particularly Z2, Z3, and Z7, demonstrated increased flexibility, indicating enhanced conformational stability upon ligand binding. These results support the potential of Z2, Z3, and Z7 as promising inhibitors that can effectively stabilize the enzyme’s active site, similar to the known inhibitor tubercidin.

### 3.6. Protein Compactness Analysis

**Figure 5:**
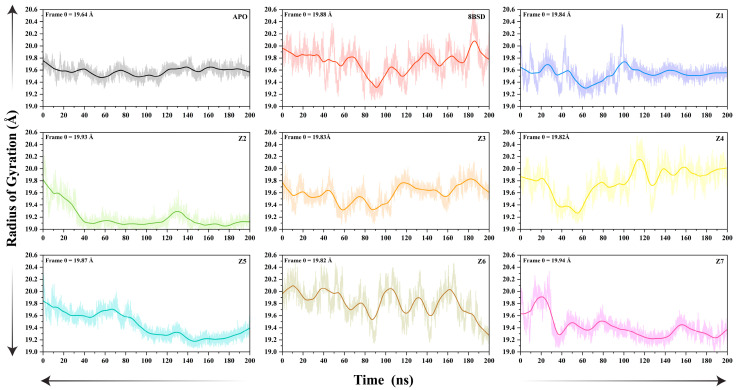
Radius of gyration (Rg) plots of SARS-CoV-2 nsp10-16 methyltransferase systems during a 200-nanosecond MD simulation, which show the compactness of the unbound enzyme (apo), the native complex with tubercidin (8BSD), and the seven inhibitor-bound systems (Z1–Z7) over simulation time. The initial Rg values (Frame 0) are explicitly marked for each system to provide a reference for structural deviations throughout the simulation.

The radius of gyration (Rg) of the SARS-CoV-2 nsp10-16 methyltransferase was analyzed over a 200-nanosecond MD simulation for the unbound (apo), native (8BSD), and inhibitor-bound systems (Z1–Z7) to assess the compactness of the protein structure during the simulation. The Rg values reflect the distribution of atoms around the protein’s center of mass, providing insight into the stability and folding of the enzyme in various states. The Rg plots for all systems are shown in Figure 5. In the apo form, the Rg values fluctuated between 19.4 Å and 20.0 Å throughout the simulation, with a significant trend toward stabilization. This indicates a relatively low degree of flexibility and structural expansion in the absence of a bound ligand, likely due to the lack of constraints in the active site region. The fluctuating Rg values suggest that the enzyme undergoes transient conformational changes, resulting in periodic expansion and contraction of the protein structure.

In contrast, the 8BSD complex exhibited more fluctuating Rg values, ranging from 19.2 Å to 20.0 Å, with fewer large fluctuations compared to the apo system. This suggests that the binding of tubercidin promotes structural compactness and stabilizes the overall conformation of the methyltransferase. The reduced variation in Rg values at the simulation’s end indicates that the presence of a ligand in the active site restricts large conformational changes, maintaining the protein in a more compact state. Among the inhibitor-bound systems, Z2, Z3, and Z7 demonstrated the lowest Rg fluctuations, with values stabilizing around 19.0 Å to 19.8 Å, similar to the native 8BSD complex. This indicates that these inhibitors effectively maintain the compactness of the methyltransferase, likely by stabilizing key structural elements near the active site. Notably, Z2 and Z7 exhibited the most consistent Rg values throughout the simulation, suggesting enhanced structural stability upon binding. Z1, Z4, Z5, and Z6 showed slightly higher Rg fluctuations at the simulation’s start compared to Z2, Z3, and Z7, with values ranging between 19.4 Å and 20.0 Å. Although the fluctuations were higher than those observed in the native complex, they were still significantly lower than those in the apo system, indicating that ligand binding contributes to some degree of structural stabilization. Among these, Z6 showed the highest Rg variation, indicating moderate flexibility in the presence of this inhibitor. Overall, the Rg analysis highlights that ligand binding plays a critical role in maintaining the compactness and structural stability of the nsp10-16 methyltransferase. The apo system showed the opposite structural expansion and flexibility, while the native complex and inhibitor-bound systems, particularly Z2, Z3, and Z7, demonstrated better structural integrity. These results suggest that Z2, Z3, and Z7 not only stabilize the active site but also help maintain the overall compactness of the protein, making them strong candidates for further experimental validation.

### 3.7. Protein Dominant Motions via Principal Component Analysis (PCA)

**Figure 6:**
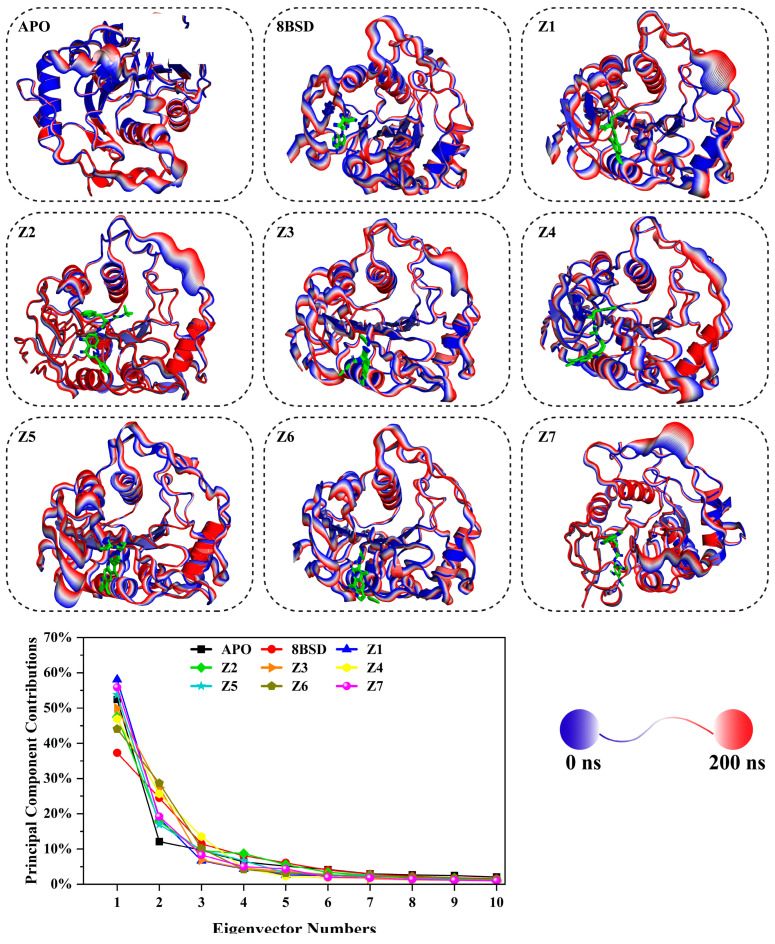
Principal component analysis (PCA) of SARS-CoV-2 nsp10-16 methyltransferase systems during a 200-nanosecond MD simulation. Cartoon representations of dominant motions captured by the first principal component (PC1) for the unbound (apo), native complex with tubercidin (8BSD), and the seven inhibitor-bound systems (Z1–Z7) are shown. The red-to-blue color gradient indicates the amplitude of movement, with red representing high motion and blue representing low motion. The ligands are included within the active pocket (green) to provide a clear reference for their spatial positioning and influence on protein dynamics. The percentage of motion contributed by the first 10 principal components is plotted for all systems.

Principal component analysis (PCA) was performed to investigate the dominant motions of the SARS-CoV-2 nsp10-16 methyltransferase during the 200-nanosecond MD simulation. The flexible N- and C-terminal residues of nsp10 and nsp16 were excluded from the analysis to focus on the core regions involved in ligand binding. The percentage of motion contributed by the first principal component (PC1) for the unbound enzyme (apo), the native complex with tubercidin (8BSD), and the seven inhibitor-bound systems (Z1–Z7) was calculated and is presented in Figure 6. PC1 represents the largest-amplitude motions in the protein, with higher percentages indicating greater flexibility and lower percentages reflecting restricted motion due to ligand binding. Among all systems, Z1 showed the highest percentage of motion (58.1%), indicating significant flexibility in its conformation during the simulation. This suggests that while Z1 binds effectively, it may not fully stabilize the enzyme’s dynamic behavior, allowing for large-scale structural motions. Z7 and Z5 followed, with percentages of motion of 55.85% and 53.79%, respectively, indicating moderate flexibility. In contrast, the 8BSD complex exhibited the lowest percentage of motion (37.3%), demonstrating that tubercidin binding significantly stabilizes the protein, minimizing large conformational changes. Among the remaining inhibitor-bound systems, Z6, Z2, Z4, and Z3 showed intermediate percentages of motion, ranging from 44.07% to 49.9%, indicating moderate stabilization. Notably, Z6 had a percentage of motion close to that of the 8BSD complex (44.07%), suggesting a better stabilizing effect compared to other inhibitors. The apo form displayed a relatively high percentage of motion (52.45%), consistent with the earlier observations of high flexibility and conformational fluctuation in the absence of a ligand. The corresponding cartoon representations of dominant motions for each system, shown in Figure 6, visually depict the conformational changes over the course of the simulation. In these representations, the red and blue color gradient indicates the amplitude of movement, with red regions indicating higher motion and blue regions indicating lower motion. In the apo system, large-scale motions were observed throughout the structure, particularly in the flexible loop regions and the active site vicinity. This is consistent with the high PC1 percentage observed for the apo form, reflecting significant flexibility and conformational changes. For the 8BSD complex, the red regions are notably smaller and more localized, indicating reduced motion throughout the enzyme. This supports the observation that tubercidin binding restricts large-scale structural rearrangements, maintaining the enzyme in a relatively stable conformation. Similar patterns were observed for inhibitor-bound systems Z1, Z3, Z4, and Z6, where the red regions were smaller compared to the apo form, suggesting that these inhibitors reduce flexibility and help maintain structural stability. Among these, Z6 displayed the least red regions, correlating with its low PC1 percentage (44.07%) and indicating that it effectively stabilizes the enzyme. In contrast, systems Z2, Z5, and Z7 exhibited larger red regions in the active site and flexible loop areas, reflecting higher conformational motions. Despite their strong binding to the active site, these inhibitors allow for greater structural fluctuations, as indicated by their relatively high PC1 percentages. The observed dominant motions for these systems suggest that while they interact effectively with key residues, they may not fully restrict the enzyme’s dynamic behavior. Overall, the PCA results reveal that the native complex with tubercidin (8BSD) provided the highest stabilization by minimizing large-scale motions. Among the inhibitors, Z6, Z2, and Z4 demonstrated better motion restriction, with PC1 percentages close to that of the 8BSD complex. These results suggest that these inhibitors could provide effective stabilization of the nsp10-16 methyltransferase—similar to the native ligand tubercidin—and support their potential as promising candidates for further experimental validation.

**Figure 7:**
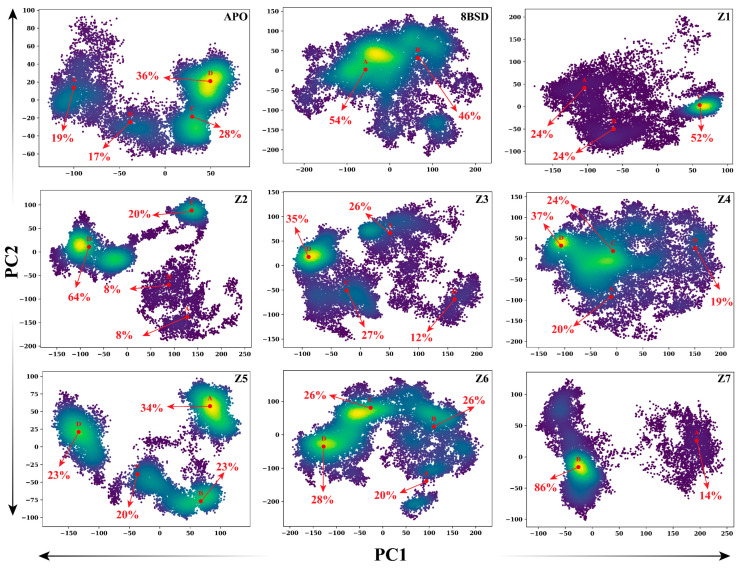
Principal component analysis (PCA) of SARS-CoV-2 nsp10-16 methyltransferase complexes highlighting conformational transitions during a 200-nanosecond MD simulation. The 2D PCA projections onto PC1 and PC2 illustrate the distribution of conformational clusters for the apo form, native complex (8BSD), and inhibitor-bound systems (Z1–Z7). The red arrows represent transitions between major conformational states, with cluster percentages indicating the population of each conformational state.

To further investigate the conformational transitions of the SARS-CoV-2 nsp10-16 methyltransferase during the 200-nanosecond MD simulation, principal component analysis (PCA) was performed to project the motions of each system onto the first two principal components (PC1 and PC2). The 2D PCA plots for the unbound enzyme (apo), the native complex with tubercidin (8BSD), and the seven inhibitor-bound systems (Z1–Z7) are presented in Figure 7, illustrating the distribution of conformational clusters sampled during the simulation. The red arrows indicate transitions between major conformational clusters, while the corresponding percentages represent the population of each cluster. In the apo system, four distinct conformational clusters were identified, with the largest cluster accounting for 36% of the total population. The transitions between clusters were frequent and widespread, indicating significant conformational flexibility and multiple structural rearrangements in the absence of a ligand. The highly dispersed nature of the apo PCA plot reflects the enzyme’s intrinsic flexibility and dynamic behavior when unbound.

The 8BSD complex exhibited two major conformational clusters, with the most populated cluster contributing 46% of the total population. Compared to the apo form, the conformational transitions in the 8BSD complex were more restricted, as indicated by the smaller spread and fewer transitions between clusters. This reduced flexibility and stabilization of specific conformations can be attributed to tubercidin binding, which restricts large-scale structural rearrangements. Among the inhibitor-bound systems, Z7 displayed two primary clusters, with one dominant cluster representing 86% of the total population. The minimal transitions and high population of a single cluster suggest that Z7 binding effectively stabilizes the enzyme, restricting conformational diversity and maintaining a more rigid structure. Similarly, Z2 showed one dominant cluster accounting for 64% of the population, indicating that Z2 also restricts large conformational transitions, akin to the 8BSD complex. In contrast, Z1, Z3, and Z4 exhibited more dispersed conformational clusters, with the dominant clusters contributing 52%, 35%, and 37%, respectively. These systems showed higher conformational transitions compared to Z2, Z6, and Z7, suggesting moderate flexibility in the enzyme when bound to these inhibitors. Z5 and Z6 demonstrated relatively balanced conformational populations, with their dominant clusters contributing 34% and 28%, respectively, indicating intermediate behavior between high flexibility and stabilization. The flipping over conformations observed in the PCA plots reveal that ligand binding significantly affects the conformational landscape of the nsp10-16 methyltransferase. The apo system showed the highest degree of conformational diversity and frequent transitions, while the native complex and inhibitor-bound systems, particularly Z2, Z4, Z6, and Z7, exhibited more restricted transitions and stabilized conformational clusters. Among the inhibitors, Z7 provided the greatest stabilization by maintaining a dominant conformation with minimal transitions, followed closely by Z2 and Z1, suggesting that these inhibitors may effectively reduce the enzyme’s conformational flexibility, similar to tubercidin. These results further support the potential of Z2, Z6, and Z7 as promising inhibitors with strong stabilizing effects on the nsp10-16 methyltransferase.

### 3.8. Hydrogen Bond Analysis

The hydrogen bond analysis plays a critical role in stabilizing protein–ligand interactions and influencing the binding affinity and structural integrity during molecular dynamics (MD) simulations (Table S7). In the native 8BSD complex, key residues involved in hydrogen bonding include Asp99, Cys115, and Tyr132—which form dominant interactions—with additional minor contributions from Asp114, Leu100, Gly71, Lys170, Asn101, and Asp130. These residues are essential for maintaining the stability of ligand binding. For the compounds Z1–Z7, several residues are consistently involved in hydrogen bonding but with varying degrees of bond lifetimes. In the Z1 complex, Asp99 and Asn101 form the strongest bonds, contributing significantly to ligand stability. Transient interactions are observed with Tyr132, Cys115, and Leu100. In Z2, Asp99 and Tyr132 play key roles, with Cys115, Leu100, Gln304, and Asn101 forming additional, weaker bonds. For Z3, significant interactions are observed with Asn101, Asp99, and Asp75, while Tyr132, Gly73, and Lys135 contribute transiently. Z4 features dominant hydrogen bonding with Asp99, Asn101, and Tyr132, with additional interactions from Cys115, Ser74, and Phe303. In Z5, Asp99, Asp102, and Ser74 are the primary contributors to ligand stabilization, with weaker bonds from Cys115, Asn101, and Tyr132. Z6 exhibits strong and long-lasting interactions with Tyr132 and Cys115, which significantly stabilize the ligand; other residues, including Asp133, Lys135, and Gly73, form weaker bonds. For Z7, key hydrogen bond interactions are formed with Asp99, Tyr132, and Cys115, with transient bonding observed with Asn101, Ser74, and Asp75. Overall, the analysis highlights Asp99, Cys115, Tyr132, and Asn101 as the critical residues involved in ligand stabilization across all complexes. Among the inhibitors, Z6 exhibited the most robust hydrogen bonding network, particularly with Tyr132 and Cys115, suggesting that it has the most stable interaction with the enzyme’s active site. The consistent involvement of Asp99 and Tyr132 across all complexes further emphasizes their pivotal role in maintaining ligand binding and the structural integrity of the protein–ligand complex. The observed interactions with Asp99 and Tyr132 suggest a potential role in ligand specificity; however, a comparative analysis with other SAM-dependent methyltransferases would be required to confirm their unique contribution to selectivity.

### 3.9. Binding Free Energy Calculations

The binding free energies of SARS-CoV-2 nsp10-16 methyltransferase complexes with tubercidin (8BSD) and the seven selected inhibitors (Z1–Z7) were calculated using the Molecular Mechanics/Generalized Born Surface Area (MM/GBSA) method, providing insights into the overall stability of the protein–ligand complexes (Table 2). Among the tested inhibitors, Z4 exhibited the most favorable total binding free energy (ΔGTOTAL = −37.81 ± 0.12 kcal/mol), followed closely by Z6 (−36.44 ± 0.06 kcal/mol), Z5 (−35.41 ± 0.07 kcal/mol), and Z7 (−34.37 ± 0.08 kcal/mol). These values indicate that these inhibitors form highly stable complexes with the methyltransferase, showing binding affinities that are comparable to or even better than the native ligand tubercidin (8BSD), which had a ΔGTOTAL of −23.90 ± 0.06 kcal/mol. This suggests that several of the selected inhibitors, particularly Z4, Z6, Z5, and Z7, exhibit superior binding stability compared to the reference ligand. Interestingly, the binding free energies of Z7, Z6, and Z5 were significantly more negative than that of 8BSD, indicating a higher binding affinity. The MM/GBSA analysis highlights these compounds as the most promising inhibitors, with Z4 emerging as the top candidate due to its most negative ΔGTOTAL. The analysis also supports the role of key residues such as Asp99, Tyr132, and Cys115 in stabilizing the ligand binding, consistent with previous hydrogen bond analyses. These residues were critical across all complexes, reinforcing their importance in the binding interactions. Additionally, water-mediated interactions were considered in the molecular dynamics simulations. While the docking simulations were performed without explicit water molecules, their inclusion in the MD simulations revealed that water molecules contribute to stabilizing the complexes, particularly for tubercidin (8BSD), which binds through several water-mediated contacts. These interactions, although not captured during docking, provide further stability to the protein–ligand binding in the full MD simulations, highlighting their significance in the binding process.

**Table 2:** MM/GBSA binding free energy analysis of SARS-CoV-2 nsp10-16 methyltransferase complexes. The energy components—including van der Waals energy (ΔE_VDW_), electrostatic energy (ΔE_EL_), polar solvation energy (ΔE_GB_), non-polar solvation energy (ΔE_SASA_), gas-phase binding energy (ΔG_GAS_), solvation free energy (ΔG_SOLV_), and total binding free energy (ΔG_TOTAL_)—are displayed for the native complex (8BSD) and inhibitor-bound systems (Z1–Z7).

Complex		MM-GBSA Calculations (All in kcal/mol)Differences (Complex–Receptor–Ligand)					
	ΔE_VDW_	ΔE_EL_	ΔE_GB_	ΔE_SASA_	ΔG_GAS_	ΔG_SOLV_	ΔG_TOTAL_
8BSD	−28.90 ± 0.06	−54.25 ± 0.61	63.01 ± 0.12	−3.75 ± 0.002	−82.95 ± 0.22	59.35 ± 0.17	−23.90 ± 0.06
Z1	−32.57 ± 0.07	−54.47 ± 0.22	59.04 ± 0.17	−4.54 ± 0.004	−86.04 ± 0.016	54.49 ± 0.12	−32.55 ± 0.04
Z2	−39.78 ± 0.05	−42.44 ± 0.10	55.43 ± 0.07	−5.44 ± 0.011	−83.23 ± 0.12	49.99 ± 0.08	−32.24 ± 0.07
Z3	−38.83 ± 0.07	−22.42 ± 0.27	29.93 ± 0.24	−3.74 ± 0.008	−59.26 ± 0.19	26.19 ± 0.18	−35.06 ± 0.05
Z4	−49.70 ± 0.11	−37.20 ± 0.47	53.32 ± 0.22	−3.87 ± 0.007	−86.71 ± 0.61	49.45 ± 0.49	−37.81 ± 0.12
Z5	−42.08 ± 0.12	−22.18 ± 0.34	33.30 ± 0.36	−4.68 ± 0.009	−63.27 ± 0.32	28.62 ± 0.36	−35.41 ± 0.07
Z6	−36.54 ± 0.09	−32.76 ± 0.22	37.53 ± 0.34	−4.44 ± 0.010	−68.31 ± 0.34	33.08 ± 0.29	−36.44 ± 0.06
Z7	−48.13 ± 0.07	−50.47 ± 0.10	69.59 ± 0.18	−5.34 ± 0.010	−99.61 ± 0.14	64.24 ± 0.12	−34.37 ± 0.08

ΔE_VDW_, van der Waals free energy; ΔE_EL_, electrostatic free energy; ΔE_PB_, the polar component of solvation-free energy; ΔE_SASA_, non-polar components of solvation energy; ΔG_GAS_, binding free energy without solvent; ΔG_SOLV_, binding free energy with solvent; ΔG_TOTAL_, total binding free energy.

### 3.10. Limitations

While our study provides valuable insights into the inhibition of SARS-CoV-2 nsp10-16 methyltransferase, certain aspects require further exploration. The findings are based on computational analyses, including molecular docking, molecular dynamics simulations, and MM/GBSA binding energy calculations. Although these approaches are well-established for predicting protein–ligand interactions, experimental validation through biochemical assays, structural studies (like X-ray crystallography or cryo-EM), and antiviral evaluations is necessary to confirm the inhibitory potential of the selected compounds. Additionally, While ADMET predictions suggest favorable pharmacokinetic properties, in vivo studies are required to assess metabolic stability, bioavailability, and potential toxicity. Furthermore, lead optimization may enhance the binding affinity, specificity, and drug-like properties to improve therapeutic potential. Despite these considerations, our study offers a robust computational framework for identifying and characterizing inhibitors of nsp10-16, laying the foundation for further experimental investigations and drug development efforts.

## 4. Conclusions

The SARS-CoV-2 nsp10-16 methyltransferase plays a vital role in viral mRNA capping, making it a promising target for antiviral intervention. This study employed structure-based drug discovery, integrating virtual screening, molecular docking, and 200-nanosecond molecular dynamics (MD) simulations to identify inhibitors. The selected seven inhibitors (Z1–Z7) exhibited strong binding affinities, with Z2, Z3, and Z7 demonstrating the most stable interactions. Molecular simulations confirmed that these inhibitors stabilized the enzyme by reducing conformational fluctuations, maintaining structural compactness, and restricting large-scale transitions. The MM/GBSA binding free energy analysis identified Z7, Z6, and Z4 as the most potent inhibitors, surpassing the native ligand tubercidin. The hydrogen bond analysis highlighted Asp99, Tyr132, and Cys115 as key residues contributing to ligand stabilization, forming persistent interactions throughout the simulation. Furthermore, ADMET profiling identified Z7 as the most promising candidate, displaying high gastrointestinal absorption, optimal solubility, and minimal CYP450 inhibition, supporting its favorable pharmacokinetic properties. These findings strongly suggest that the inhibition of nsp10-16 could serve as a viable antiviral strategy against SARS-CoV-2. Future studies should focus on experimental validation, enzymatic inhibition assays, and lead optimization to enhance drug-like properties for clinical applications.

## Figures and Tables

**Table 1:** Docking scores and RMSD-refined values for the seven selected ZINC compounds targeting the SARS-CoV-2 nsp10-16 methyltransferase.

Code	ZINC ID	SMILES	Docking Score (kcal/mol)	RMSD Refined
Z1	ZINC544166	O=C(O)[C@@](NC(=O)[C@@](NC(=O)CCC=1C(=O)Oc2c(C=1C)cc1c(C)c(C)oc1c2)C)Cc1c2c([nH]c1)cccc2	−8.15	3.63
Z2	ZINC2087193	O=C(NCCc1c2c([nH]c1)ccc(OC)c2)Nc1c(C(=O)OC)cc(OC)c(OC)c1	−8.08	1.50
Z3	ZINC2109321	O=C(N[C@](C(=O)O)c1ccccc1)/C(/NC(=O)c1ccccc1)=C/c1cc(OC)c(OC)c(OC)c1	−8.08	1.49
Z4	ZINC2111032	O=C(OC(C)(C)C)N[C@](C(=O)Oc1cc(O)c2C(=O)C=C(c3ccccc3)Oc2c1)Cc1c2c([nH]c1)cccc2	−7.97	1.28
Z5	ZINC2111034	Fc1ccc(-c2c3c(c(C)c4OC(=O)C(CC(=O)N[C@](C(=O)O)CCCNC(=O)N)=C(C)c4c3)oc2)cc1	−7.87	2.34
Z6	ZINC2112495	O=C(O)[C@](NC(=O)[C@@](NC(=O)CCC=1C(=O)Oc2c(C=1C)cc1c(C)c(C)oc1c2)C)Cc1c2c([nH]c1)cccc2	−7.56	3.09
Z7	ZINC2112958	O=C(N[C@](C(=O)O)Cc1ccccc1)Nc1c(OC)cc(OC)cc1	−7.40	3.62
8BSD	tubercidin	OCC1C(O)C(O)C(n2ccc3c(N)ncnc23)O1	−6.62	0.51

## Data Availability

Data is contained within the article and Supplementary Materials.

## Supplementary Materials

The following supporting information can be downloaded at https://www.mdpi.com/article/10.3390/cimb47030198/s1.
