# Supplementary material for: Structure-Based Identification of SARS-CoV-2 nsp10-16 Methyltransferase Inhibitors Using Molecular Dynamics Insights
Source: Curr Issues Mol Biol. 2025 Mar 17;47(3):198. doi: 10.3390/cimb47030198 (PMC11941477; doi:10.3390/cimb47030198)
Supplement: Supplementary file 1 [file cimb-47-00198-s001.zip › cimb-3480585-supplementary.pdf]

Structure-Based Identification of SARS-CoV-2 nsp10-16 Methyltransferase Inhibitors Using Molecular Dynamics Insights

Ahmad M. Alharbi [1*]

[1] Department of Clinical Laboratories Sciences, College of Applied Medical Sciences, Taif University, P.O. Box 11099, Taif 21944, Saudi Arabia.

* Correspondence: a.alharbii@tu.edu.sa

Table S1. Detailed docking interactions of the final seven compounds (Z1–Z7) from the ZINC20 in-stock database.

Compounds	2D structure	Ligand Atoms	Receptor Atoms	Residues	Bond Type	Distance (Å)	Energy (kcal/mol)
8BSD	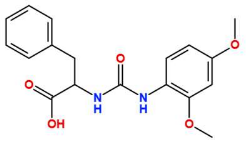	O5	O	TYR132	HBD	2.5	-2.0
		O2	OD1	ASP99	HBD	2.5	-4.0
		O3	OD2	ASP99	HBD	2.7	-3.6
		N1	N	CYS115	HBA	3.0	-4.0
		6-ring	CB	LEU100	pi-H	3.9	-0.5
Z1	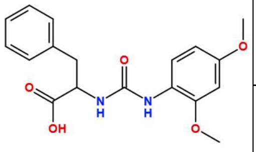	N7	OD1	ASP99	HBD	2.9	-3.3
		N10	OD1	ASP99	HBD	3.4	-0.5
		N10	OD2	ASP99	HBD	2.9	-6.6
		O21	O	TYR132	HBD	2.6	-6.8
		O9	N	TYR132	HBA	2.9	-4.6
		O20	ND2	ASN101	HBA	2.9	-1.1
Z2	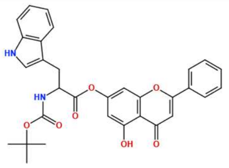	N8	O	TYR132	HBD	2.9	-1.8
		N13	OD1	ASP99	HBD	2.8	-4.2
		O26	OD2	ASP99	HBD	2.6	-1.0
		C37	OD2	ASP114	HBD	3.3	-0.5

		O29	N	CYS115	HBA	3.2	-2.6
		6-ring	N	PHE149	pi-H	4.4	-0.6
Z3	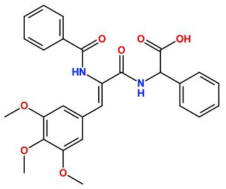	N	O	TYR132	HBD	3.4	-1.5
		N	OD1	ASP99	HBD	3.1	-6.2
		O	N	CYS115	HBA	3.0	-0.6
		O	ND2	ASN101	HBA	3.1	-0.8
		6-ring	CD	PRO134	pi-H	3.8	-0.5
Z4	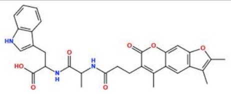	N20	OD1	ASP99	HBD	3.2	-2.6
		N20	OD2	ASP99	HBD	2.8	-5.2
		N25	O	TYR132	HBD	2.9	-1.7
		O34	OD2	ASP99	HBD	2.5	-5.6
		O19	N	TYR132	HBA	3.3	-2.3
Z5	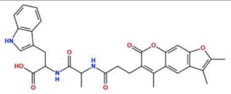	N15	OD1	ASP99	HBD	3.1	-2.0
		N15	OD2	ASP99	HBD	2.8	-5.8
		N20	OD1	ASP99	HBD	3.5	-1.3
		N25	O	TYR132	HBD	2.8	-3.4
		O34	O	SER74	HBD	2.7	-0.8
		O9	N	CYS115	HBA	3.4	-0.7

		O14	N	TYR132	HBA	2.9	-6.1
		C36	6-ring	PHE149	H-pi	4.1	-0.6
Z6	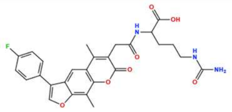	N7	OD2	ASP99	HBD	2.8	-6.9
		N12	OD1	ASP99	HBD	3.0	-3.5
		O18	O	TYR132	HBD	2.7	-5.3
		O17	ND2	ASN101	HBA	2.8	-2.7
		O20	N	CYS115	HBA	2.9	-4.2
		5-ring	N	PHE149	pi-H	4.2	-1.2
Z7	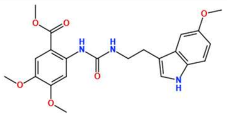	N15	OD1	ASP99	HBD	2.8	-4.7
		N15	OD2	ASP99	HBD	3.1	-1.0
		N18	OD2	ASP99	HBD	2.8	-5.8
		N23	O	TYR132	HBD	2.9	-1.7
		O4	ND2	ASN101	HBA	2.9	-3.3
		O17	CA	MET131	HBA	3.2	-0.6
		O17	N	TYR132	HBA	3.1	-3.8

Table S2. Physicochemical properties and structural characteristics of the final seven compounds (Z1–Z7) from the ZINC20 in-stock database, detailing molecular weight, atom counts, and functional groups.

Molecule	Canonical SMILES	Formula	MW	Heavy atoms	Aromatic heavy atoms	Fraction Csp3	Rotatable bonds
Z1	O=C(N[C](C(=O)N[C](C(=O)O)Cc1c[nH]c2c1cccc2)C)CCc1c(=O)oc2c(c1C)cc1c(c2)oc(c1C)C	C31H29N3O7	555.58	41	22	0.29	11
Z2	COc1ccc2c(c1)c(CCNC(=O)Nc1cc(OC)c(cc1C(=O)OC)OC)c[nH]2	C22H25N3O6	427.45	31	15	0.27	11
Z3	COc1cc(/C=C(/C(=O)N[C](c2ccccc2)C(=O)O)\NC(=O)c2ccccc2)cc(c1OC)OC	C27H25N2O7	489.5	36	18	0.15	12
Z4	O=C(OC(C)(C)C)N[C](C(=O)Oc1cc(O)c2c(c1)oc(cc2=O)c1ccccc1)Cc1c[nH]c2c1cccc2	C31H27N2O7	539.56	40	25	0.19	10
Z5	O=C(Cc1c(=O)oc2c(c1C)cc1c(c2C)occ1c1ccc(cc1)F)N[C](C(=O)O)CCCNC(=O)N	C27H25FN3O7	522.5	38	19	0.26	11
Z6	O=C(N[C](C(=O)N[C](C(=O)O)Cc1c[nH]c2c1cccc2)C)CCc1c(=O)oc2c(c1C)cc1c(c2)oc(c1C)C	C31H29N3O7	555.58	41	22	0.29	11
Z7	COc1cc(OC)ccc1NC(=O)N[C](C(=O)O)Cc1ccccc1	C18H19N2O5	343.35	25	12	0.22	9

Table S3. Drug-likeness and pharmacophore assessment of compounds Z1–Z7 based on Lipinski, Ghose, Veber, Egan, and Muegge rules, including PAINS and Brenk alerts.

Molecule	Lipinski violations	Ghose violations	Veber violations	Egan violations	Muegge violations	PAINS alerts	Brenk alerts
Z1	1	2	2	1	1	0	1
Z2	0	0	1	0	0	0	0
Z3	0	2	1	0	0	0	1
Z4	1	2	0	0	1	0	1
Z5	1	2	2	1	1	0	1

Z6	1	2	2	1	1	0	1
Z7	0	0	0	0	0	0	0

Table S4. Solubility predictions and absorption profiles for compounds Z1–Z7, including ESOL, Ali, and Silicos-IT Log S values, gastrointestinal absorption, and blood-brain barrier permeability.

Molecule	ESOL Log S	Ali Log S	Silicos-IT LogSw	GI absorption	BBB permeant
Z1	-5.46	-6.92	-9.89	Low	No
Z2	-4.22	-5.34	-7.02	High	No
Z3	-4.89	-6.15	-7.16	High	No
Z4	-6.76	-8.5	-9.82	Low	No
Z5	-4.38	-5.74	-8.55	Low	No
Z6	-5.46	-6.92	-9.89	Low	No
Z7	-3.27	-4.13	-5.01	High	No

Table S5. Lipophilicity and synthetic accessibility of compounds Z1–Z7, featuring multiple LogP estimates and consensus LogP values.

Molecule	iLOGP	XLOGP3	WLOGP	MLOGP	Silicos-IT Log P	Consensus Log P	Synthetic Accessibility
Z1	0	3.97	3.95	1.7	6.2	3.16	4.89
Z2	3.57	3.33	3.15	1.5	3.34	2.98	3.3
Z3	0	3.87	2.87	1.78	3.46	2.4	4.12
Z4	0	5.98	5.57	2.33	5.64	3.9	4.57
Z5	0	2.63	3.82	1.97	4.6	2.6	4.61
Z6	0	3.97	3.95	1.7	6.2	3.16	4.89
Z7	0	2.45	2.21	1.9	1.82	1.68	2.94

Table S6. Cytochrome P450 enzyme inhibition potential of compounds Z1–Z7, with specific focus on CYP1A2, CYP2C19, CYP2C9, CYP2D6, and CYP3A4.

Molecule	CYP1A2 inhibitor	CYP2C19 inhibitor	CYP2C9 inhibitor	CYP2D6 inhibitor	CYP3A4 inhibitor
Z1	Yes	No	Yes	No	No
Z2	No	Yes	Yes	Yes	Yes
Z3	No	Yes	Yes	Yes	No
Z4	No	No	Yes	No	No
Z5	No	No	No	No	No

Z6	Yes	No	Yes	No	No
Z7	No	No	No	No	No

Table S7. Comprehensive analysis of hydrogen bond interactions for compounds 8BSD and Z1–Z7 with key residues in the nsp10-16 methyltransferase, including bond life percentages, distances, and angles, demonstrating the stability and specificity of each ligand-receptor interaction.

Compounds	Acceptor Atoms	Donor Atoms	Bond Life	Bond Distance (Å)	Bond Angle (°)
8BSD	TBN-421-N1	CYS-115-N	54.65%	2.91	157.61
	ASP-99-OD1	TBN-421-O2'	52.64%	2.68	160.37
	ASP-99-OD2	TBN-421-O3'	49.74%	2.68	165.09
	ASP-99-OD2	TBN-421-O2'	44.55%	2.69	160.54
	ASP-99-OD1	TBN-421-O3'	40.70%	2.68	164.38
	ASP-114-OD2	TBN-421-N6	25.82%	2.84	160.18
	TYR-132-O	TBN-421-O5'	24.89%	2.75	156.34
	ASP-114-OD2	TBN-421-N6	17.85%	2.84	160.09
	ASP-114-OD1	TBN-421-N6	16.05%	2.84	160.37
	ASP-130-O	TBN-421-O5'	15.60%	2.73	154.61
	ASP-114-OD1	TBN-421-N6	13.12%	2.84	160.23
	TBN-421-O2'	ASN-101-ND2	6.42%	2.90	155.85
	TBN-421-N3	LEU-100-N	5.07%	2.93	145.46
	TBN-421-O4'	TYR-132-N	1.66%	2.92	159.76
	TBN-421-O5'	TYR-132-N	0.86%	2.91	144.63
	TBN-421-O2'	LEU-100-N	0.48%	2.93	155.16
	GLY-71-O	TBN-421-O5'	0.26%	2.83	158.58
	TBN-421-O5'	LYS-170-NZ	0.24%	2.91	155.36
	TBN-421-O5'	LYS-170-NZ	0.23%	2.91	155.58
	TBN-421-O5'	LYS-170-NZ	0.14%	2.90	155.93
	ASP-130-OD1	TBN-421-O5'	0.07%	2.77	156.97
	TBN-421-O3'	ASN-101-ND2	0.06%	2.88	161.24
	ASP-130-OD2	TBN-421-O5'	0.04%	2.76	160.34
Z1	ASP-99-OD1	Z1-421-O21	54.11%	2.63	157.04
	ASP-99-OD2	Z1-421-N10	43.33%	2.84	152.87
	ASP-99-OD2	Z1-421-O21	37.55%	2.63	157.26
	ASP-99-OD2	Z1-421-N7	34.73%	2.86	152.33
	ASP-99-OD1	Z1-421-N10	32.51%	2.84	153.15
	ASP-99-OD1	Z1-421-N7	21.33%	2.87	152.35
	Z1-421-O20	ASN-101-ND2	19.03%	2.89	152.79
	Z1-421-O21	ASN-101-ND2	13.75%	2.91	159.40
	Z1-421-O21	ASN-101-N	3.25%	2.91	145.81

	Z1-421-O9	TYR-132-N	3.21%	2.87	154.85
	ASP-130-O	Z1-421-O21	0.58%	2.70	158.46
	Z1-421-O2	CYS-115-N	0.58%	2.91	161.52
	Z1-421-O20	TYR-132-N	0.16%	2.90	158.80
	Z1-421-O23	LEU-100-N	0.06%	2.90	141.94
	Z1-421-O20	ASN-101-ND2	0.00%	2.82	143.17
	Z1-421-O21	LEU-100-N	0.00%	2.95	135.69
**Z2**	ASP-99-OD1	Z2-421-O26	51.12%	2.61	163.64
	ASP-99-OD2	Z2-421-O26	48.38%	2.63	164.57
	Z2-421-O21	TYR-132-N	47.70%	2.88	158.62
	ASP-99-OD2	Z2-421-N13	43.19%	2.80	160.64
	ASP-99-OD1	Z2-421-N13	40.76%	2.80	161.12
	TYR-132-O	Z2-421-N8	2.06%	2.86	154.11
	Z2-421-O29	CYS-115-N	1.74%	2.92	159.74
	Z2-421-O26	LEU-100-N	1.48%	2.94	157.08
	Z2-421-O29	GLN-304-NE2	0.13%	2.87	152.85
	Z2-421-O38	GLN-304-NE2	0.02%	2.93	153.37
	Z2-421-O38	TYR-132-N	0.01%	2.94	142.51
	Z2-421-N13	ASN-101-ND2	0.01%	2.98	144.97
**Z3**	Z3-421-O10	ASN-101-ND2	58.61%	2.86	159.84
	ASP-99-OD1	Z3-421-N19	43.11%	2.85	154.16
	ASP-99-OD2	Z3-421-N19	44.70%	2.87	148.85
	ASP-99-OD2	Z3-421-O23	44.10%	2.62	159.06
	ASP-99-OD1	Z3-421-O23	23.10%	2.62	160.05
	ASP-75-OD2	Z3-421-O23	17.60%	2.64	160.44
	Z3-421-O18	ASN-101-ND2	14.50%	2.85	160.82
	GLY-73-O	Z3-421-O23	9.30%	2.72	159.93
	ASP-75-OD1	Z3-421-O23	7.20%	2.64	158.66
	TYR-132-O	Z3-421-N19	6.70%	2.87	155.54
	Z3-421-O18	ASN-101-ND2	5.20%	2.88	148.57
	Z3-421-O32	TYR-132-N	3.50%	2.90	147.20
	Z3-421-O22	ASP-75-N	2.90%	2.91	145.11
	Z3-421-O10	ASN-101-ND2	0.22%	2.88	156.58
	Z3-421-O32	LYS-135-NZ	0.20%	2.87	147.69
	Z3-421-O32	LYS-135-NZ	0.19%	2.90	148.96
	Z3-421-O32	LYS-135-NZ	0.18%	2.88	150.81
	GLY-71-O	Z3-421-O23	0.09%	2.71	152.13
	Z3-421-O35	LYS-135-NZ	0.08%	2.87	153.81
	Z3-421-O35	LYS-135-NZ	0.07%	2.89	149.19
	Z3-421-O35	LYS-135-NZ	0.06%	2.90	150.21
	ASN-101-OD1	Z3-421-N19	0.03%	2.86	146.45

	Z3-421-O22	LYS-135-NZ	0.02%	2.80	159.57
	Z3-421-O2	CYS-115-N	0.02%	2.92	144.24
	SER-74-O	Z3-421-O23	0.02%	2.74	159.74
	Z3-421-O23	TYR-132-N	0.02%	2.85	160.15
	Z3-421-O22	ASN-101-ND2	0.02%	2.88	149.81
	Z3-421-O23	GLY-73-N	0.02%	2.92	138.92
	Z3-421-O23	ASP-75-N	0.02%	2.95	146.21
	ASP-133-OD2	Z3-421-O23	0.01%	2.70	150.28
	Z3-421-O23	ASN-101-ND2	0.01%	2.84	145.76
	Z3-421-O22	TYR-132-N	0.01%	2.85	140.29
	Z3-421-O22	ASN-101-ND2	0.01%	2.87	154.68
	Z3-421-O23	LYS-135-NZ	0.01%	2.90	161.43
	Z3-421-O35	CYS-115-N	0.01%	2.90	146.94
	Z3-421-O22	LYS-170-NZ	0.01%	2.91	140.48
	Z3-421-O2	LEU-100-N	0.01%	2.94	139.79
**Z4**	ASP-99-OD2	Z4-421-O34	53.39%	2.62	156.29
	Z4-421-O33	ASN-101-ND2	29.92%	2.88	156.42
	ASP-99-OD1	Z4-421-O34	29.80%	2.62	155.35
	ASP-99-OD1	Z4-421-N20	18.63%	2.87	160.58
	Z4-421-O14	TYR-132-N	12.17%	2.89	156.37
	ASP-99-OD2	Z4-421-N20	10.07%	2.84	151.92
	Z4-421-O34	ASN-101-ND2	8.50%	2.91	156.77
	Z4-421-O34	ASN-101-N	7.29%	2.92	151.50
	ASP-130-O	Z4-421-N25	6.07%	2.83	158.80
	Z4-421-O14	ASN-101-ND2	3.09%	2.86	154.13
	ASP-99-OD2	Z4-421-N25	1.36%	2.82	153.33
	Z4-421-O14	CYS-115-N	1.33%	2.90	162.30
	Z4-421-O19	TYR-132-N	1.01%	2.90	160.37
	Z4-421-O3	PHE-303-N	0.90%	2.91	159.26
	Z4-421-O33	ASN-101-N	0.73%	2.91	152.94
	Z4-421-O9	LYS-135-NZ	0.68%	2.84	150.54
	Z4-421-O9	LYS-135-NZ	0.64%	2.84	152.96
	Z4-421-O9	LYS-135-NZ	0.55%	2.83	149.72
	ASP-99-OD1	Z4-421-N25	0.44%	2.83	155.97
	GLY-71-O	Z4-421-N25	0.38%	2.83	149.11
	ASN-101-OD1	Z4-421-O34	0.26%	2.78	149.38
	ASP-130-OD1	Z4-421-N25	0.13%	2.85	148.03
	ASN-101-OD1	Z4-421-N20	0.12%	2.86	153.02
	Z4-421-O33	SER-74-N	0.11%	2.90	149.51
	ASP-130-OD2	Z4-421-N25	0.08%	2.81	155.38
	TYR-132-O	Z4-421-N25	0.06%	2.83	150.08

	Z4-421-O3	LYS-135-NZ	0.06%	2.92	155.17
	Z4-421-O3	LYS-135-NZ	0.05%	2.93	154.75
	Z4-421-O34	TYR-132-N	0.04%	2.94	161.16
	Z4-421-O3	LYS-135-NZ	0.03%	2.93	159.49
	GLY-73-O	Z4-421-N25	0.03%	2.88	149.49
	ASP-102-O	Z4-421-N25	0.02%	2.88	157.87
	Z4-421-O3	ASN-298-ND2	0.02%	2.92	157.63
	Z4-421-O9	PHE-149-N	0.02%	2.95	161.32
	Z4-421-O3	ASN-101-ND2	0.02%	2.97	142.54
	Z4-421-O7	GLN-304-NE2	0.01%	2.92	146.30
	ASN-101-ND2	Z4-421-N20	0.01%	2.95	144.13
	SER-74-O	Z4-421-N25	0.01%	2.86	158.96
	Z4-421-O7	LYS-135-NZ	0.01%	2.92	140.67
	Z4-421-O7	LYS-135-NZ	0.01%	2.95	142.58
**Z5**	ASP-99-OD2	Z5-421-N15	52.44%	2.83	157.04
	ASP-99-OD1	Z5-421-N15	47.20%	2.86	154.51
	ASP-102-O	Z5-421-O34	34.70%	2.76	157.10
	ASP-99-OD1	Z5-421-O34	34.00%	2.66	158.22
	ASP-99-OD2	Z5-421-N20	31.20%	2.86	154.02
	Z5-421-O33	SER-74-N	27.20%	2.90	151.33
	Z5-421-O33	ASN-101-ND2	7.79%	2.86	157.72
	Z5-421-O3	CYS-115-N	3.72%	2.92	151.82
	ASP-99-OD1	Z5-421-N20	1.73%	2.88	157.95
	ASN-101-O	Z5-421-O34	1.72%	2.76	146.79
	ASP-99-OD2	Z5-421-O34	1.30%	2.70	156.52
	Z5-421-O9	LEU-100-N	1.23%	2.89	155.11
	Z5-421-O33	ASN-101-ND2	0.94%	2.86	156.45
	ASP-75-OD1	Z5-421-O34	0.62%	2.63	159.69
	Z5-421-O34	ASN-101-ND2	0.56%	2.90	155.97
	Z5-421-O14	TYR-132-N	0.53%	2.89	159.44
	Z5-421-O33	ASP-102-N	0.46%	2.93	159.16
	Z5-421-O34	ASN-101-N	0.44%	2.90	147.77
	TYR-132-O	Z5-421-N25	0.38%	2.87	156.20
	ASP-75-O	Z5-421-N25	0.31%	2.85	145.94
	Z5-421-O34	SER-74-N	0.29%	2.93	150.00
	ASP-75-OD2	Z5-421-O34	0.20%	2.66	149.32
	ASP-75-OD2	Z5-421-N25	0.18%	2.81	154.53
	Z5-421-O34	ASN-101-ND2	0.18%	2.90	153.35
	Z5-421-O14	LYS-135-NZ	0.13%	2.82	150.51
	Z5-421-O14	LYS-135-NZ	0.12%	2.84	151.82
	SER-74-O	Z5-421-O34	0.11%	2.75	153.59

	SER-74-O	Z5-421-N25	0.08%	2.92	146.09
	Z5-421-O9	GLY-71-N	0.08%	2.90	141.48
	ASP-102-O	Z5-421-N25	0.07%	2.84	153.18
	ASP-75-OD1	Z5-421-N25	0.07%	2.85	149.47
	Z5-421-O34	ASP-75-N	0.06%	2.93	147.08
	Z5-421-O34	ASP-102-N	0.05%	2.96	150.96
	Z5-421-O14	LYS-135-NZ	0.02%	2.87	146.53
	Z5-421-O34	LEU-100-N	0.02%	2.94	144.91
	Z5-421-O33	LYS-135-NZ	0.01%	2.84	147.34
	Z5-421-O33	LYS-135-NZ	0.01%	2.70	141.44
	Z5-421-O34	GLY-77-N	0.01%	2.88	150.33
	Z5-421-O33	ASP-75-N	0.01%	2.90	157.44
**Z6**	TYR-132-O	Z6-421-N7	80.25%	2.84	157.61
	Z6-421-O20	CYS-115-N	74.40%	2.91	162.35
	TYR-132-O	Z6-421-O18	71.97%	2.74	155.11
	ASP-133-OD2	Z6-421-N14	4.50%	2.85	155.02
	ASP-99-OD1	Z6-421-N7	3.65%	2.83	165.29
	ASP-133-OD1	Z6-421-N14	3.35%	2.86	155.66
	ASP-133-OD2	Z6-421-N12	3.15%	2.88	155.46
	ASP-133-OD1	Z6-421-N12	2.36%	2.87	155.34
	Z6-421-O15	LYS-135-NZ	1.95%	2.82	155.43
	Z6-421-O15	LYS-135-NZ	1.82%	2.80	155.11
	Z6-421-O15	ASN-101-ND2	1.82%	2.86	158.70
	Z6-421-O15	LYS-135-NZ	1.76%	2.82	155.40
	TYR-132-O	Z6-421-N12	1.36%	2.87	155.08
	Z6-421-O17	ASN-101-ND2	1.26%	2.84	157.64
	ASP-99-OD2	Z6-421-N14	0.68%	2.86	153.62
	TYR-132-O	Z6-421-N14	0.58%	2.89	150.64
	ASP-99-OD1	Z6-421-N14	0.56%	2.88	156.23
	Z6-421-O17	ASN-101-ND2	0.56%	2.89	158.21
	ASP-99-OD1	Z6-421-N12	0.52%	2.90	156.51
	GLY-73-O	Z6-421-N14	0.36%	2.88	147.26
	Z6-421-O15	ASN-298-ND2	0.16%	2.90	159.56
	Z6-421-O17	LYS-135-NZ	0.10%	2.83	154.06
	ASP-75-OD1	Z6-421-N14	0.10%	2.87	155.25
	ASP-99-OD2	Z6-421-N12	0.09%	2.91	150.79
	ASP-75-OD2	Z6-421-N14	0.08%	2.88	153.31
	Z6-421-O18	ASN-101-ND2	0.08%	2.89	150.80
	Z6-421-O26	TYR-152-OH	0.08%	2.89	155.85
	Z6-421-O15	ASN-101-ND2	0.08%	2.90	158.94
	ASP-133-OD1	Z6-421-O18	0.07%	2.63	161.38

	ASN-101-OD1	Z6-421-N14	0.07%	2.86	154.22
	SER-74-O	Z6-421-N14	0.07%	2.90	149.67
	Z6-421-O17	LYS-135-NZ	0.07%	2.85	148.78
	ASP-133-OD2	Z6-421-O18	0.06%	2.63	159.60
	Z6-421-O17	LYS-135-NZ	0.05%	2.84	156.48
	Z6-421-O6	GLN-304-NE2	0.05%	2.81	157.93
	ASP-133-OD2	Z6-421-N7	0.03%	2.81	164.40
	GLY-71-O	Z6-421-N14	0.03%	2.86	146.87
	Z6-421-F33	LYS-135-NZ	0.02%	2.74	148.65
	TYR-302-O	Z6-421-N12	0.02%	2.85	160.46
	Z6-421-O17	ASN-101-N	0.02%	2.89	142.19
	Z6-421-F33	LYS-135-NZ	0.02%	2.87	144.92
	Z6-421-O18	LYS-135-NZ	0.02%	2.91	146.16
	Z6-421-O21	CYS-115-N	0.02%	2.97	151.77
**Z7**	ASP-99-OD2	Z7-421-N15	56.91%	2.76	160.74
	Z7-421-O17	TYR-132-N	42.34%	2.89	160.12
	ASP-99-OD1	Z7-421-N15	40.56%	2.76	160.40
	ASP-99-OD2	Z7-421-N18	23.78%	2.88	148.02
	ASP-99-OD1	Z7-421-N18	14.81%	2.88	148.18
	Z7-421-O8	CYS-115-N	14.56%	2.92	160.50
	Z7-421-O11	CYS-115-N	2.70%	2.88	142.40
	Z7-421-O4	ASN-101-ND2	0.54%	2.86	153.70
	Z7-421-O2	ASN-101-ND2	0.30%	2.89	154.36
	Z7-421-O28	SER-74-OG	0.22%	2.81	159.66
	Z7-421-O28	ASP-75-N	0.17%	2.92	150.72
	Z7-421-O28	ASN-43-ND2	0.13%	2.89	154.79
	ASP-99-OD1	Z7-421-N23	0.10%	2.82	141.34
	TYR-132-O	Z7-421-N23	0.02%	2.82	148.57
	ASP-75-OD2	Z7-421-N23	0.02%	2.82	145.67
	ASP-75-OD1	Z7-421-N23	0.02%	2.88	157.14
	ASP-99-OD2	Z7-421-N23	0.01%	2.79	137.74
	SER-74-O	Z7-421-N23	0.01%	2.86	146.65
